# Creatine-electrolyte supplementation improves repeated sprint cycling performance: A double blind randomized control study

**DOI:** 10.1186/s12970-018-0226-y

**Published:** 2018-05-02

**Authors:** Daniel L. Crisafulli, Harsh H. Buddhadev, Lorrie R. Brilla, Gordon R. Chalmers, David N. Suprak, Jun G. San Juan

**Affiliations:** Kinesiology Program, Department of Health and Human Development, Western Washington University, Carver 201L, MS 9067, 516 High Street, Bellingham, WA 98225 USA

**Keywords:** Creatine, Sprint cycling, Recovery interval, Sprint duration, Ergometer

## Abstract

**Background:**

Creatine supplementation is recommended as an ergogenic aid to improve repeated sprint cycling performance. Furthermore, creatine uptake is increased in the presence of electrolytes. Prior research examining the effect of a creatine-electrolyte (CE) supplement on repeated sprint cycling performance, however, did not show post-supplementation improvement. The purpose of this double blind randomized control study was to investigate the effect of a six-week CE supplementation intervention on overall and repeated peak and mean power output during repeated cycling sprints with recovery periods of 2 min between sprints.

**Methods:**

Peak and mean power generated by 23 male recreational cyclists (CE group: *n* = 12; 24.0 ± 4.2 years; placebo (P) group: *n* = 11; 23.3 ± 3.1 years) were measured on a Velotron ergometer as they completed five 15-s cycling sprints, with 2 min of recovery between sprints, pre- and post-supplementation. Mixed-model ANOVAs were used for statistical analyses.

**Results:**

A supplement-time interaction showed a 4% increase in overall peak power (pre: 734 ± 75 W; post: 765 ± 71 W; *p* = 0.040; η_p_^2^ = 0.187) and a 5% increase in overall mean power (pre: 586 ± 72 W; post: 615 ± 74 W; *p* = 0.019; η_p_^2^ = 0.234) from pre- to post-supplementation for the CE group. For the P group, no differences were observed in overall peak (pre: 768 ± 95 W; post: 772 ± 108 W; *p* = 0.735) and overall mean power (pre: 638 ± 77 W; post: 643 ± 92 W; *p* = 0.435) from pre- to post-testing. For repeated sprint analysis, peak (pre: 737 ± 88 W; post: 767 ± 92 W; *p* = 0.002; η_p_^2^ = 0.380) and mean (pre: 650 ± 92 W; post: 694 ± 87 W; *p* < 0.001; η_p_^2^ = 0.578) power output were significantly increased only in the first sprint effort in CE group from pre- to post-supplementation testing. For the P group, no differences were observed for repeated sprint performance.

**Conclusion:**

A CE supplement improves overall and repeated short duration sprint cycling performance when sprints are interspersed with adequate recovery periods.

## Background

Creatine is widely recommended as an ergogenic aid to improve performance during short bouts of high-intensity physical activity, such as cycling sprints [1–5]. Supplementation with creatine increases an individual’s total muscle creatine content (i.e. intramuscular phosphocreatine and creatine) [6–11]. Research supports that increased intramuscular phosphocreatine increases the capacity of the phosphagen system to provide rapid energy turnover during cycling sprints; thus, leading to increased power production while delaying the onset of fatigue [1–5]. Furthermore, the post-supplementation increase in intramuscular creatine availability increases the rate that phosphocreatine is resynthesized following the cessation of exercise [7, 9, 10], potentially leading to improved performance during subsequent sprints. Consequently, previous studies have investigated the effects of creatine supplementation on peak and mean power output during individual [12–14] and repeated sprint cycling tests [6, 10, 12, 15–27].

The composition of the supplementation material plays a critical role in creatine uptake and the post-supplementation effect on power generating capabilities [28]. The majority of previous studies examining creatine supplementation and sprint cycling performance have used creatine monohydrate [6, 12, 14–16, 18–21, 23–27, 29]. It is important to note, however, that creatine uptake is increased in the presence of electrolytes [30–34]. Creatine transport is primarily dependent on electrogenic transporter proteins, requiring at least two sodium ions and one chloride ion to transport one creatine molecule across a cellular membrane [30–33]. Results of the studies by Dai et al. [30] and Peral et al. [32] demonstrate when the extracellular concentration of creatine remains constant, the rate and magnitude of creatine uptake is increased with increasing concentrations of extracellular sodium and chloride. Interestingly, Dai et al. [30] also reported compared to the control condition creatine uptake was decreased by 47% when calcium and magnesium were absent from the extracellular fluid. The creatine-electrolyte supplement used in the present study contains these electrolytes (i.e. sodium, chloride, calcium, and magnesium).

Results of the study by Stout et al. [28] demonstrate an improved ergogenic effect of creatine supplementation when the supplement composition included electrolytes. Stout et al. [28] compared the effects of 8 weeks of supplementation with creatine and electrolytes (CE; 5.25 g creatine monohydrate, 633 mg of sodium and potassium phosphates, 33 g glucose, and 1 g taurine) versus creatine monohydrate (CM; 5.25 g creatine monohydrate and 1 g glucose) on bench press one-repetition maximum (1RM), vertical jump height, and 100-yard dash time. Subjects in this study consisted of male NCAA Division II football players who participated in an identically structured strength/power/speed training program during the supplementation intervention. For all performance measures, Stout et al. [28] found significantly greater improvement in the CE compared to CM group. For bench press 1RM, improvements from pre- to post-supplementation were 12.9% and 7.3%, for the CE and CM groups, respectively. For vertical jump height, improvements were 8.4% and 3.1% for the CE and CM groups, respectively. For the 100-yard dash, improvements (i.e. shown by reduction in time) were − 2.6% and − 2.4% for the CE and CM groups, respectively. These findings suggest that the ergogenic effect of creatine supplementation is enhanced when the creatine substance is taken in combination with electrolytes.

With regards to creatine-electrolyte supplementation and repeated sprint cycling performance, however, previous researchers have not reported significant pre- to post-supplementation increases in peak power output, and have reported mixed results with respect to changes in mean power output [35, 36]. While it is difficult to speculate why improvements in peak power output were not found in these studies, aspects of the sprint cycling protocol, namely the sprint and recovery durations, may partly explain the mixed results with respect to post-supplementation changes in mean power output. For example, it is likely that the relatively long sprint and short recovery durations (i.e. 20 s each) utilized by Finn et al. [35] would have resulted in extensive intramuscular phosphocreatine depletion during the sprints, and would not have allowed for adequate intramuscular phosphocreatine resynthesis during the recovery periods [37–39]. This may have decreased the sensitivity of the testing protocol at detecting post-supplementation changes in mean power output during the first and subsequent sprints.

For sprint durations lasting 6–10 s, phosphocreatine levels deplete to 55–57% of resting levels when measured immediately post-sprint, and the phosphagen system contributes about 50% of the overall energy requirement of the sprint [4, 37, 40]. With an increase in sprint duration to 20 and 30 s, the depletion of intramuscular phosphocreatine reaches 73% and 83%, respectively, and the overall contribution of the phosphagen system to the energy demands of the sprint decreases to about 25% [4, 37–39]. Sprint durations of 20–30 s rely heavily on the glycolytic energy system, thus, decreasing the efficacy of the test to elicit performance enhancements post-creatine supplementation [37–39]. Therefore, sprint durations of 20–30 s may not be appropriate for examining the capacity of the phosphocreatine system. In order to ideally examine the capacity of the phosphagen system during sprint cycling, investigators Cottrell, Coast, and Herb [18] suggest employing sprint durations of 15 s.

Inter-sprint recovery duration is also a crucial factor when assessing the phosphagen system because phosphocreatine is resynthesized during recovery [7, 10, 37, 38]. Following a protocol consisting of electrically evoked contractions of the leg extensors, previously shown to result in near total muscle phosphocreatine degradation, Greenhaff et al. [7] reported that the initial 20 s of a two-minute passive recovery period contributed only about 31% of the total intramuscular phosphocreatine resynthesis in the vastus lateralis. Furthermore, Greenhaff et al. [7] reported that there were no significant differences in the pre- and post-supplementation rates of intramuscular phosphocreatine resynthesis during the first minute of passive recovery. However, the post-supplementation rate of phosphocreatine resynthesis was significantly increased during the second minute of passive recovery [7]. Additionally, Bogdanis et al. [34] reported that in a non-supplemented state, following 10- and 20-s sprints, recovery intervals of 2 min allowed the resynthesis of phosphocreatine to about 86% and 76% resting levels, respectively. These findings suggest that an inter-sprint recovery interval of 2 min would increase the recovery of intramuscular phosphocreatine content when compared to shorter recovery periods, and increase the sensitivity of the cycling sprint testing protocol to detect post-supplementation performance improvements. However, no research has investigated the efficacy of creatine-electrolyte supplementation on repeated sprint cycling performance when 15-s sprints were interspersed with 2 min of passive recovery. Sprint cycling peak and mean power outputs attenuate with repeated sprints [40, 41]. Kreider et al. [36], who previously investigated the ergogenic effect of creatine-electrolyte supplementation for 12 repeated sprints, only found improvements in the first five sprints. It is for this reason five sprints were chosen for the current study. Therefore, the purpose of this study was to investigate the effect of a six-week creatine-electrolyte supplementation intervention on overall and repeated peak and mean power output during repeated short duration sprint cycling performance, in a group of recreational cyclists. We hypothesized that 6 weeks of creatine and electrolyte supplementation would result in significant increases in overall and repeated peak and mean power output during repeated short duration sprint cycling performance when the sprint and recovery durations were 15 s and 2 min, respectively.

## Methods

### Study design

This was a randomized double-blind placebo controlled study. Before completing pre-supplementation testing, participants were randomly assigned to either the creatine-electrolyte (CE) or placebo (P) group. Pre-supplementation testing was followed by a six-week intervention period, during which participants were supplemented with either the CE or P material, depending on their group assignment. Subjects then completed post-supplementation testing, which consisted of identical testing procedures as pre-supplementation testing. Differences in peak and mean power output during sprint cycling were examined from pre- to post-supplementation testing and between the CE and P groups.

### Subjects

Twenty-five male recreational cyclists, between the ages of 19–33 years, who self-reported riding a bicycle or indoor trainer at a moderate to vigorous intensity for at least 1 h twice per week over the past 6 months, were recruited for this study. Moderate to vigorous intensity is defined as fairly light to very hard, or a rating of perceived exertion of 12–17 on a 6–20 scale [42]. Based on a statistical power analysis, a total sample size of 16 participants (eight per group) was needed to achieve a statistical power of 0.8 to detect a large effect size for supplement-time (i.e. within-between groups) interaction at an alpha level of 0.05. The sample size computation was based on the study by Flanagan and Jakeman [43]. This study was chosen for power analysis because they contrasted the effects of creatine supplementation versus placebo on repeated sprint cycling peak power output in young men. They found large effect sizes (i.e. partial eta squared (η_p_^2^)) for peak power output variables across repeated sprints ranging from 0.215–0.602. We were conservative in our statistical power analysis and used an effect size of η_p_^2^ = 0.15 for the sample size computation. The η_p_^2^ = 0.15 was chosen because Vincent [44] reported effect sizes over 0.15 to be large in magnitude.

Subjects were screened for conditions that could affect creatine absorption or metabolism, or limit their ability to perform cycling sprints. These conditions included known creatine metabolism or transportation deficiencies; cardiac, kidney, liver, or spleen disease/dysfunction; and any musculoskeletal injuries or neuromuscular conditions (upper and lower body, head, neck, or trunk) that would cause pain or discomfort, or limit a subject’s ability to cycle comfortably during sprint testing [4, 45, 46]. Furthermore, subjects who had supplemented with creatine within the previous 60 days were excluded from the study. Thirteen participants in the CE group and 12 participants in the P group completed pre-supplementation testing. One participant from each group did not complete post-supplementation testing, and were dropped from the study. The participant in the P group did not complete post-supplementation testing because he sustained a knee injury unrelated to the study. The participant in the CE group experienced gastrointestinal discomfort and withdrew himself from the study. The final participant pool consisted of 23 recreational male cyclists, 12 in the CE group, and 11 in the P group. Only data from participants who completed pre- and post-supplementation testing were included in data analysis. The demographics of the subjects who completed pre- and post-supplementation testing are presented in Table 1.

**Table 1:** Subject demographic characteristics

	Creatine-Electrolyte	Placebo
*n*	12	11
Age (years)	24.0 ± 4.2	23.3 ± 3.1
Mass (kg)	71.8 ± 5.2	75.4 ± 10.1
Height (m)	1.75 ± 0.04	1.79 ± 0.09

Values are mean ± one standard deviation

### Data collection

Prior to participation in the study, subjects were provided with and signed an informed consent form, which had previously been approved by the University’s Institutional Review Board. Before each testing session, subjects were asked to: refrain from lower body exercise and consuming alcohol at least 24 h prior to testing; refrain from any exercise and consuming caffeine at least 4 h prior to testing; drink approximately 500 ml of water 2 h prior to testing; and empty their bladder and bowel immediately prior to testing. Upon arrival for pre-supplementation testing, subjects completed a medical history form. After confirming the subjects met the medical criteria for participation, they were provided with standardized clothing, which included clipless cycling shoes, cycling shorts, and a top. Each subject’s mass and height were measured on a standard balance beam scale with stadiometer (Detecto, Webb City, MO).

During sprint cycling testing, an electronically-braked Velotron cycle ergometer (Racer-Mate Inc., Seattle, WA) interfaced with its corresponding Velotron Wingate software (Racer-Mate Inc., Seattle, WA) was used to measure peak and mean power outputs at a sampling frequency of 10 Hz. The Velotron cycle ergometer is a reliable instrument for measuring power output during sprint cycling [47, 48], and has been previously used to investigate variables of sprint cycling [49, 50]. In order to control cycling posture, which is known to affect energy cost [51], joint ranges of motion and muscle activation patterns [52], a standardized bike fitting procedure was performed. Seat height and fore/aft position were adjusted so that when the pedal surface was parallel to the ground, and the subject’s pedal was at the bottom of the pedal stroke (6 o’clock), their knee was in a position of 25–30° of flexion [53, 54]. Furthermore, when the crank arms were parallel to the ground (3 o’clock), a plumb line dropped from the inferior pole of the patella of the more forward knee dissected the pedal spindle [54]. Handle bar height and fore/aft position were adjusted so that when the subjects placed their hands on the brake hoods and maintained a slight flexion in their elbows, their trunk angle was equal to 30° of flexion with respect to the vertical [55, 56].

Following the bike fitting procedure, subjects completed a five-minute warm-up on the ergometer at a self-selected cadence and resistance. Three minutes of passive recovery, during which the subjects remained seated on the Velotron, separated the end of the warm-up and beginning of the sprint cycling testing protocol. The subjects then completed a total of five 15-s sprints, each interspersed with 2 min of passive recovery. The sprints were performed at the subject’s maximal cadence against a flywheel resistance relative to the subject’s body mass (0.075 kp per kg body mass), a commonly used relative resistive load [14, 38, 48, 50, 57]. Immediately before each 15-s sprint, prior to applying resistance to the flywheel, subjects were allowed 3 s to increase the flywheel speed from a standstill, providing a flying start. Equivalent verbal encouragement was provided to all participants during each sprint. Post-supplementation testing followed identical procedures as pre-supplementation testing, and was completed within 3 days of the final day of terminating the supplementation intervention.

In order to compare the CE and P groups’ macronutrient and total energy intake, as well as energy expenditure, subjects completed a three-day diet record (two weekdays and one weekend day) and the Bouchard Three-Day Physical Activity Record [58] during the same 3 days over the supplementation intervention. Three-day diet records were subsequently entered into Nutritionist Pro software (Axxya Systems, Stafford, TX), which was used to determine macronutrient and total energy intake. Subjects were asked to keep their training programs and diets consistent throughout the study.

### Supplementation protocol

After being randomly assigned to either the CE or P group, and after completing pre-supplementation testing, the subjects were provided with their respective supplementation material. Each subject was given a package containing 42 individually packaged daily doses (i.e. one tablespoon each) of his respective supplementation material, which lasted the duration of the intervention. The CE group consumed 4 g of creatine combined with electrolytes (i.e. 114 mg sodium chloride, 171 mg calcium chloride, 286 mg magnesium chloride, and 171 mg potassium chloride) per day. The placebo group consumed a placebo treatment of an equal volume of maltodextrin per day, a commonly used placebo material [19, 21, 59, 60]. All subjects were instructed to orally consume one packaged dose per day with approximately 500 ml of water. All subjects were given a 28 fluid oz. shaker bottle (BlenderBottle Company, Lehi, UT) and 42 sugar and caffeine free Orange Crush instant drink packets (The Jel Sert Company, West Chicago, IL), which they were allowed to mix with their supplementation material for improved palatability. If a subject reported missing more than three total supplement days, they were dropped from the study. No subjects were dropped from the study for this reason.

### Statistical analysis

Overall peak power output was defined as the maximum power output identified by the Wingate software across any one of the five 15-s cycling sprints. Overall mean power output was the average power output maintained across all five sprints. Repeated peak and mean power output refers to the peak and mean power output of each individual sprint. Two-way mixed model ANOVAs with repeated measures on time (i.e. pre- and post-supplementation) were used to assess the effects of supplementation (i.e. CE and P) on overall peak and mean power output, and body mass. Three-way mixed model ANOVAs with repeated measures on time and sequential sprints (i.e. pre- and post-supplementation and sprints 1–5) were used to assess the effects of supplementation on repeated peak and mean power output. Alpha level was set a priori at *p* < 0.05. For significant supplement-time interactions, post-hoc simple effects analyses were performed with t-tests. Prior to conducting ANOVAs, the data were checked for normality, homogeneity of variance, and sphericity using the Shapiro-Wilk test, Levene’s test of equality of error variance, and Mauchly’s test of sphericity, respectively. When the assumption of sphericity of data was violated a Greenhouse-Geisser correction was applied to the alpha level. In addition, the effect size was calculated as partial eta squared (η_p_^2^). Partial eta squared was interpreted in accordance with the guidelines provided by Vincent [44], where, η_p_^2^ > 0.01 was small, η_p_^2^ > 0.06 was medium, and η_p_^2^ > 0.15 was large. Descriptive data are provided as means and standard deviations. All statistical procedures were performed using SPSS (Version 23).

## Results

**Fig. 1:**
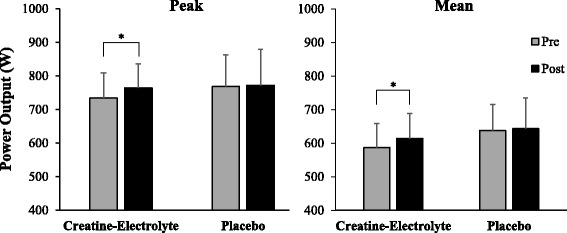
Overall peak and mean power output (W) during the five 15-s sprints in the CE and P groups, pre- and post-supplementation. * Indicates significant improvement in sprint performance from pre- to post-testing (*p* < 0.05)

For all dependent variables the assumptions of normality and homogeneity of variance were not violated. When the assumption of sphericity of data was violated a Greenhouse-Geisser correction was applied to the alpha level. After 6 weeks of supplementation, significant supplement-time interactions of large effect sizes were observed for overall peak power (*F*_*1,22*_ = 4.820, *p* = 0.040, η_p_^2^ = 0.187) and overall mean power output (*F*_*1,22*_ = 6.432, *p* = 0.019, η_p_^2^ = 0.234). Post-hoc comparisons revealed that there were no significant differences in overall peak or mean power outputs between the CE and P groups during pre-supplement testing. From pre- to post-supplementation testing, there were significant increases in overall peak and mean power output during the five 15-s sprints when supplemented with creatine-electrolytes, but not when supplemented with the placebo treatment. Overall peak power output during the five 15-s sprints increased by 4.16% (30.50 ± 33.31 W; *p* = 0.002) for the CE group, compared to 0.40% (3.09 ± 25.65 W; *p* = 0.735) for the P group. Similarly, overall mean power output across the five 15-s sprints increased by 4.82% (28.29 ± 16.73 W; *p* < 0.001) for the CE group compared to 0.82% (5.22 ± 26.25 W; *p* = 0.435) for the P group. The overall peak and mean power output data are shown in Fig. 1.

**Fig. 2:**
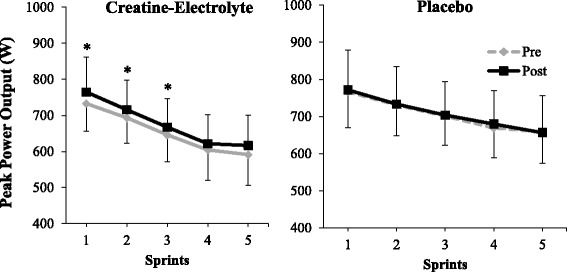
Peak power output (W) during each of the five 15-s sprints for the CE and P group, pre- and post-supplementation. * Indicates significant improvement in sprint performance from pre- to post-testing (*p* < 0.05)

We conducted further statistical analysis to discern pre- to post-supplementation changes in repeated peak and mean power output per sprint for the CE versus P group. For the peak power output, no statistical differences were found for a three-way interaction (supplement x time x sprint: *p* = 0.590) and two-way interactions (supplement x sprint: *p* = 0.191; time x sprint: *p* = 0.0842). For the two-way supplement x time interaction a non-significant statistical trend was observed for the peak power output data (*p* = 0.076; η_p_^2^ = 0.149). A significant main effect for time was also observed indicating 2% increase in peak power output from pre- to post-testing (*p* = 0.020; η_p_^2^ = 0.243). No differences were observed between the groups for the main effect of supplement (*p* = 0.304). For the time x supplement interaction, post-hoc comparisons were conducted to determine the changes in repeated peak power output from pre- to post-testing between the CE and P groups (Fig. 2). For the CE group, peak power output was 3–4% greater from pre- to post-supplementation for the first three sprints (*p* < 0.05; η_p_^2^ > 0.208); whereas, for the P group, no changes were observed in peak power output over any sprint (*p* > 0.05; η_p_^2^ < 0.048).

**Fig. 3:**
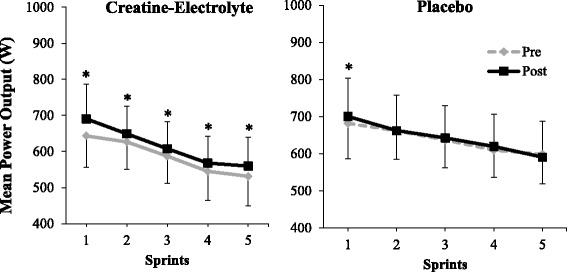
Mean power output (W) during each of the five 15-s sprints in the CE and P group, pre- and post-supplementation. * Indicates significant improvement in sprint performance from pre- to post-testing (*p* < 0.05)

For the mean power output, no statistical differences were found for a three-way interaction (supplement x time x sprint: *p* = 0.587) and two way interactions (supplement x sprint: *p* = 0.229; time x sprint: *p* = 0.136). However, the two-way supplement x time interaction was statistically significant for the mean power output data (*p* = 0.025; η_p_^2^ = 0.226). A significant main effect for time was also observed indicating a 2.7% increase in mean power output from pre- to post-testing (*p* = 0.002; η_p_^2^ = 0.381). For the main effect of supplement no differences were observed between the groups (*p* = 0.328). For the significant time x supplement interaction, post-hoc comparisons were conducted to determine the changes in repeated mean power output from pre- to post-testing between the CE and P groups. For the CE group, mean power output improved 3–7% from pre- to post-testing for each of the sprints (*p* < 0.05; η_p_^2^ > 0.198; Fig. 3). For the P group, no improvements were observed in mean power output from pre- to post-testing with the exception of sprint 1, which showed a small (2.7%) improvement in sprint performance from pre- to post-testing (*p* = 0.043; η_p_^2^ = 0.189).

**Fig. 4:**
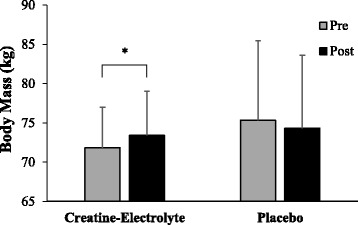
Body mass (kg) for the CE and P groups during pre- and post-supplementation. * Indicates significant changes from pre- to post-testing (*p* < 0.05)

A supplement-time interaction was also observed for body mass (*p* = 0.001; η_p_^2^ = 0.405). Post-hoc comparisons revealed a significant pre- to post-supplementation increase in body mass of 1.6 ± 1.4 kg (*p* = 0.003) for the CE group, and 1.0 ± 1.9 kg (*p* = 0.053) decrease in body mass for P group. Pre- and post-supplementation body mass data for the CE and P groups are presented in Fig. 4.

Data for dietary intake and physical activity are presented in Table 2 below. These data show that subjects in both the groups did not have significantly different diet and physical activity levels (*p* > 0.05).

**Table 2:** Physical activity and dietary intake values as assessed by three-day Bouchard and dietary records

	Creatine-Electrolyte	Placebo	*t-test p* values
Energy Expended (kcal · day^−1^)	3539 ± 285	3300 ± 579	*p* = 0.305
Energy Intake (kcal · day^− 1^)	2623 ± 921	2371 ± 499	*p* = 0.481
Dietary Carbohydrates (g · day^− 1^)	310 ± 112	266 ± 80	*p* = 0.351
Dietary Protein (g · day^− 1^)	129 ± 64	106 ± 14	*p* = 0.899
Dietary Fat (g · day^− 1^)	96 ± 32	98 ± 19	*p* = 0.320

Values are mean ± one standard deviation

## Discussion

The purpose of this study was to investigate the effect of a six-week creatine-electrolyte supplementation intervention on overall and repeated peak and mean power output during repeated short duration sprint cycling. Results of the present study support the hypothesis that creatine-electrolyte supplementation would lead to significant increases in overall and repeated peak and mean power output during repeated short duration sprint cycling performance when the sprints were interspersed with 2 min of passive recovery. We hypothesized that supplementation with the creatine-electrolyte material would lead to improved repeated sprint cycling performance, as the literature generally supports the effectiveness of creatine supplementation at improving such performance [1–4]. Supplementation with creatine increases one’s intramuscular creatine and phosphocreatine contents [6–11], which has been implicated as a contributing factor for the ergogenic effect of creatine supplementation [1–4]. Increased intramuscular phosphocreatine increases the rate and duration that the phosphagen system is able to contribute rapid energy turnover; thus, increasing peak and mean power outputs during sprint cycling [1–4]. Furthermore, the increase in intramuscular creatine content increases the rate of phosphocreatine resynthesis during recovery; thus, improving subsequent sprint performances [7, 9, 10]. Supplementation with creatine also typically increases one’s body mass [7–10, 13, 18, 19, 21, 27, 28, 35, 36, 60–63], presumably due to increased intramuscular total creatine content and the associated increases in water retention and/or lean body mass [28, 36, 62, 63]. Therefore, considering the significant improvements in overall and repeated peak and mean power outputs, and significant increase in body mass, it is reasonable to expect that the creatine-electrolyte supplement increased the intramuscular creatine and phosphocreatine concentrations of the subjects following that treatment.

Improvements in sprint cycling performance observed in our study demonstrate the expected ergogenic effect of creatine-electrolyte supplementation. These outcomes were expected because creatine monohydrate supplementation improves peak and mean power output during sprint cycling [6, 10, 12, 16, 24–26]. Electrolytes further improve creatine uptake [30–33] and the ergogenic effect [28]. Creatine transport into cells is mediated via transporter proteins, which operate in an electrogenic fashion, requiring sodium and chlorine ions. Dai et al. [30] and Peral et al. [32] reported that the rate and magnitude of creatine uptake were increased when the extracellular solution contained these electrolytes, compared to when these electrolytes were absent. With creatine monohydrate supplementation, the greatest increase in intramuscular total creatine content occurs during the initial 6–28 days of supplementation, depending on the supplementation protocol [64]. After this, intramuscular total creatine content typically levels off, demonstrating a cellular creatine saturation effect [11, 65, 66]. To the authors’ knowledge, the effect of electrolytes on muscle creatine saturation is unknown. Although cellular creatine saturation likely occurs early in the intervention, sustained supplementation with creatine results in further increases in body mass and fat free mass [62]. Stout et al. [28] contrasted the effects of creatine-electrolyte versus creatine monohydrate on anaerobic power in NCAA division II athletes. For the creatine-electrolyte group, they found significantly greater improvement in anaerobic power (i.e. bench press 1RM, vertical jump height, and 100-yard dash time) compared to the creatine monohydrate group. Taken together, these results suggest that sustained supplementation with a creatine-electrolyte material may yield greater effect than supplementing with creatine monohydrate alone. However, when Finn et al. [35] and Kreider et al. [36] investigated the ergogenic effect of creatine electrolyte supplementation, they did not observe an increase in peak power output across any of their cycling sprints. Our study is the first to demonstrate an improvement in overall and repeated peak power output across cycling sprints, post creatine-electrolyte supplementation.

In the present study, peak power output was almost always (~ 97% of trials) observed during the first sprint effort, and systematically decreased from there. Similar to the present study, peak power output demonstrated a systematic decline during subsequent sprint performances in the study by Finn et al. [35]. In the present study, overall peak power output was increased by ~ 4% in the CE group from pre- to post-supplementation testing. For sprints 1–3, peak power output was increased by 4%, 3%, and 3%, respectively (Fig. 3). Presumably due to lack of significant results, neither Finn et al. [35] nor Kreider et al. [36] report both pre- and post-supplementation peak power output values. Therefore, it is not possible to compare post-creatine-electrolyte supplementation changes in peak power output in the present study with those by Finn et al. [35] and Kreider et al. [36].

The overall mean power output sustained across all five 15-s sprints interspersed with 2 min of passive recovery by subjects in this study, regardless of group identification and testing time, was 620 ± 79 watts. Across four 20-s sprints interspersed with 20 s of passive recovery, subjects in the study by Finn et al. [35] maintained an overall mean power output of about 600 watts. Across 12 six-second sprints interspersed with 30 s of passive recovery, subjects in the study by Kreider et al. [36] sustained an overall mean power output of about 900 watts. However, due to numerous methodological factors that affect peak and mean power output, researchers must exercise caution when comparing data across supplementation and sprint cycling studies. Some of the methodological factors affecting power outputs include: cycle ergometer type [47, 48]; the subjects’ training status [26, 67], sex [68], and age [26]; and aspects of the sprint cycling protocol (i.e. resistive load applied [68, 69], starting technique [49, 70], sprint and recovery durations [39, 40], and sprint cycling posture [71, 72]).

In each of these studies, including our study, similar to peak power output, mean power output maintained per sprint systematically declined with successive sprints. In the present study, mean power output of the first sprint was 678 ± 88 watts, and decreased to 570 ± 91 watts. Finn et al. [35] reported that their subjects maintained a mean power output of about 700 watts during their first 20-s cycling sprint. Thereafter, mean power output decreased steadily to about 500 watts during the fourth 20-s sprint effort. Mean power output results (reported as total work) of the study by Kreider et al. [36] demonstrate the same decreasing pattern. In that study, subjects maintained a mean power output of about 1200 watts during the first of 12 six-second sprints interspersed with 30 s of passive recovery. During the final sprint in that study, mean power output decreased to about 800 watts.

In the present study, mean power output maintained per sprint was significantly increased by about 3–7% across all five sprints following creatine-electrolyte supplementation (Fig. 3). Finn et al. [35] did not observe pre- to post-supplementation changes in mean power output during any sprint. Kreider et al. [36] report significant pre- to post-supplementation improvements in mean power output of about 10–15% per sprint during the first five of 12 sprints for their creatine-electrolyte group. However, from sprints 6–12, the differences in pre- to post-supplementation mean power output were not significantly different between the creatine-electrolyte and placebo groups. Interestingly, in the study by Kreider et al. [36] the placebo group also demonstrated a 5–10% improvement in mean power output per sprint across all 12 sprints. Therefore, in the study by Kreider et al. [36] the performance improvements following creatine-electrolyte supplementation were about 5–10% greater than the changes shown by the placebo group. While the post-supplementation improvements in mean power output for the creatine-electrolyte and placebo groups in the study by Kreider et al. [36] appear to be large, it is important to note the study’s subject demographics. The subjects in the study by Kreider et al. [36] were NCAA division IA football players who participated in a structured exercise program consisting of 5 h per week of heavy resistance training and 3 h per week of agility and sprint training during their four-week supplementation intervention.

Results of the present study and those by Finn et al. [35] and Kreider et al. [36] emphasize the importance of the sprint and recovery durations when assessing the efficacy of creatine-electrolyte supplementation at improving repeated sprint cycling performance. However, since overall peak power output is typically recorded during the first sprint, it is unlikely that the either the sprint or recovery duration influenced overall peak power output in this study and those by researchers Finn et al. [35] and Kreider et al. [36]. Therefore, it remains unknown why subjects in the studies by Finn et al. [35] and Kreider et al. [36] did not demonstrate significantly increased peak power output when supplemented with creatine and electrolytes. For mean power output, however, the sprint and recovery durations are crucial aspects to consider for the sprint cycling protocol. When the sprint duration is equal to the duration of the recovery (1:1 work to recovery), ample resynthesis of phosphocreatine does not occur during the inter-sprint recovery [7, 9]. Thus, post-supplementation improvements in sprint performance during subsequent sprints are unlikely. Results of Finn et al. [35] demonstrate this scenario, as the duration of their sprint and recovery interval were both 20 s (1:1). Conversely, when Kreider et al. [36] utilized a shorter sprint interval (6 s) and longer recovery duration (30 s), a 1:5 work to recovery ratio, they found improved mean power output during the first five of 12 sprints. The inter-sprint recovery interval utilized in the study by Kreider et al. [36] allowed for enough phosphocreatine resynthesis to detect post-supplementation improvements in mean power output during the initial five sprints. In the present study, the inter-sprint recovery interval was longer in relation to the sprint duration (i.e. 15-s sprint and 120 s of recovery, or a 1:8 work to recovery ratio). Perhaps, this longer recovery interval allowed for greater phosphocreatine resynthesis, resulting in improved peak and mean power outputs during subsequent sprint performances.

We observed an overall 3–7% improvement in sprint cycling performance following creatine-electrolyte supplementation. These improvements are similar to improvements observed during sprint cycling testing following supplementation with creatine monohydrate alone, typically ranging between 2 and 9% [6, 10, 12, 16, 18, 24–27, 43]. On face value, there appears to be no added benefit of supplementing with a creatine-electrolyte versus creatine monohydrate material. However, due to the numerous methodological factors that influence power output during sprint cycling, it is not possible to determine if creatine-electrolyte supplementation enhances the ergogenic effect of creatine supplementation. Future research should address this by conducting a similar experiment while assessing the differences in sprint cycling performance post-supplementation with creatine-electrolytes compared to creatine monohydrate alone. Furthermore, future studies should investigate the underlying mechanisms of action for the significant post-supplementation increase in body mass, and assess the contribution of the increased body mass on the subjects’ power generating abilities.

## Conclusion

In summary, results of the present study indicate that 6 weeks of creatine-electrolyte supplementation leads to significant increases in overall and repeated peak and mean power output during repeated sprint cycling when the sprint and recovery durations are 15 s and 2 min, respectively. The increase in peak power output observed in this study is the first time a significant increase in overall and repeated peak power output has been observed during sprint cycling following creatine-electrolyte supplementation. These results suggest that recreational cyclists wanting to increase their overall and repeated peak and mean power output during repetitve sprint cycling performances involving sprint and recovery durations similar to those used in this study may benefit from participating in a creatine-electrolyte supplementation protocol similar to the one used in the present study.

## Abbreviations

CE: Creatine-electrolyte
P: Placebo

## References

[1] Bemben MG, Lamont HS (2005). Creatine supplementation and exercise performance: Recent findings. Sport Med..

[2] Mesa JLM, Ruiz JR, González-Gross MM, Gutiérrez Sáinz A, Castillo Garzón MJ (2002). Oral creatine supplementation and skeletal muscle metabolism in physical exercise. Sport Med.

[3] Demant TW, Rhodes EC (1999). Effects of creatine supplementation on exercise performance. Sport Med..

[4] Turjung RL, Clarkson P, Eichner ER, Greenhaff PL, Hespel PJ, Israel RG (2000). The physiological and health effects of oral creatine supplementation. Med Sci Sport Exerc..

[5] Kreider RB, Kalman DS, Antonio J, Ziegenfuss TN, Wildman R, Collins R (2017). International Society of Sports Nutrition position stand: Safety and efficacy of creatine supplementation in exercise, sport, and medicine. J Int Soc Sports Nutr..

[6] Ahmun RP, Tong RJ, Grimshaw PN (2005). The effects of acute creatine supplementation on multiple sprint cycling and running performance in rugby players. J Strength Cond Res..

[7] Greenhaff PL, Bodin K, Soderlund K, Hultman E (1994). Effect of oral creatine supplementation on skeletal muscle phosphocreatine resynthesis. Am J Phys.

[8] Hickner RC, Dyck DJ, Sklar J, Hatley H, Byrd P (2010). Effect of 28 days of creatine ingestion on muscle metabolism and performance of a simulated cycling road race. J Int Soc Sports Nutr.

[9] Yquel RJ, Arsac LM, Thiaudière E, Canioni P, Manier G (2002). Effect of creatine supplementation on phosphocreatine resynthesis, inorganic phosphate accumulation and pH during intermittent maximal exercise. J Sports Sci.

[10] Preen D, Dawson B, Goodman C, Lawrence S, Beilby J, Ching S (2001). Effect of creatine loading on long-term sprint exercise performance and metabolism. Med Sci Sport Exerc..

[11] Harris RC, Söderlund K, Hultman E (1992). Elevation of creatine in resting and exercised muscle of normal subjects by creatine supplementation. Clin Sci.

[12] Dawson B, Cutler M, Moody A, Lawrence S, Goodman C, Randall N (1995). Effects of oral creatine loading on single and repeated maximal short sprints. Aust J Sci Med Sport.

[13] Snow RJ, McKenna MJ, Selig SE, Kemp J, Stathis CG, Zhao S (1998). Effect of creatine supplementation on sprint exercise performance and muscle metabolism. J Appl Physiol.

[14] Odland LM, MacDougall JD, Tarnopolsky MA, Elorriaga A, Borgmann A (1997). Effect of oral creatine supplementation on muscle [PCr] and short-term maximum power output. Med Sci Sport Exerc..

[15] Barnett C, Hinds M, Jenkins DG (1996). Effects of oral creatine supplementation on multiple sprint cycle performance. Aust J Sci Med Sport.

[16] Birch R, Noble D, Greenhaff PL (1994). The influence of dietary creatine supplementation on performance during repeated bouts of maximal isokinetic cycling in man. Eur J Appl Physiol Occup Physiol.

[17] Cooke WH, Grandjean PW, Barnes WS (1995). Effect of oral creatine supplementation on power output and fatigue during bicycle ergometry. J Appl Physiol.

[18] Cottrell GT, Coast JR, Herb RA (2002). Effect of recovery interval on multiple-bout sprint cycling performance after acute creatine supplementation. J Strength Cond Res..

[19] Deutekom M, Beltman JG, de Ruiter CJ, de Koning JJ, de Haan A (2000). No acute effects of short-term creatine supplementation on muscle properties and sprint performance. Eur J Appl Physiol.

[20] Havenetidis K, Matsouka O, Cooke CB, Theodorou A (2003). The use of varying creatine regimes on sprint cycling. J Sport Sci Med.

[21] Kinugasa R, Akima H, Ota A, Ohta A, Sugiura K, Kuno SY (2004). Short-term creatine supplementation does not improve muscle activation or sprint performance in humans. Eur J Appl Physiol.

[22] Gill ND, Hall RD, Blazevich AJ (2004). Creatine serum is not as effective as creatine powder for improving cycle sprint performance in competitive male team-sport athletes. J Strength Cond Res..

[23] Okudan N, Gokbel H (2005). The effects of creatine supplementation on performance during the repeated bouts of supramaximal exercise. J Sports Med Phys Fitness.

[24] Tarnopolsky MA, MacLennan DP (2000). Creatine monohydrate supplementation enhances high-intensity exercise performance in males and females. Int J Sport Nutr Exerc Metab..

[25] Vandebuerie F, Vanden Eynde B, Vandenberghe K, Hespel P (1998). Effect of creatine loading on endurance capacity and sprint power in cyclists. Int J Sports Med.

[26] Wiroth JB, Bermon S, Andreï S, Dalloz E, Hébuterne X, Dolisi C (2001). Effects of oral creatine supplementation on maximal pedalling performance in older adults. Eur J Appl Physiol.

[27] Ziegenfuss TN, Rogers M, Lowery L, Mullins N, Mendel R, Antonio J (2002). Effect of creatine loading on anaerobic performance and skeletal muscle volume in NCAA Division I athletes. Nutrition.

[28] Stout J, Eckerson J, Noonan D, Moore G, Cullen D (1999). Effects of 8 weeks of creatine supplementation on exercise performance and fat-free weight in football players during training. Nutr Res.

[29] Jones AM, Atter T, George KP (1999). Oral creatine supplementation improves multiple sprint performance in elite ice-hockey players. J Sports Med Phys Fitness..

[30] Dai W, Vinnakota S, Qian X, Kunze DL, Sarkar HK (1999). Molecular characterization of the human CRT-1 creatine transporter expressed in Xenopus oocytes. Arch Biochem Biophys.

[31] Guimbal C, Kilimann MW (1993). A Na^+^-dependent creatine transporter in rabbit brain, muscle, heart, and kidney. J Biol Chem.

[32] Peral MJ, García-Delgado M, Calonge ML, Durán JM, De La Horra MC, Wallimann T (2002). Human, rat and chicken small intestinal Na^+^-Cl^−^-creatine transporter: Functional, molecular characterization and localization. J Physiol.

[33] Snow RJ, Murphy RM (2001). Creatine and the creatine transporter: A review. Mol Cell Biochem.

[34] Schoch RD, Willoughby D, Greenwood M (2006). The regulation and expression of the creatine transporter: A brief review of creatine supplementation in humans and animals. J Int Soc Sports Nutr..

[35] Finn JP, Ebert TR, Withers RT, Carey MF, Mackay M, Phillips JW (2001). Effect of creatine supplementation on metabolism and performance in humans during intermittent sprint cycling. Eur J Appl Physiol.

[36] Kreider RB, Ferreira M, Wilson M, Grindstaff P, Plisk S, Reinardy J (1998). Effects of creatine supplementation on body composition, strength, and sprint performance. Med Sci Sport Exerc..

[37] Bogdanis GC, Nevill ME, Lakomy HKA, Boobis LH (1998). Power output and muscle metabolism during and following recovery from 10 and 20 s of maximal sprint exercise in humans. Acta Physiol Scand.

[38] Bogdanis GC, Nevill ME, Boobis LH, Lakomy HK (1996). Contribution of phosphocreatine and aerobic metabolism to energy supply during repeated sprint exercise. J Appl Physiol.

[39] Smith JC, Hill DW (1991). Contribution of energy systems during a Wingate power test. Br J Sports Med.

[40] Gaitanos GC, Williams C, Boobis LH, Brooks S (1993). Human muscle metabolism during intermittent maximal exercise. J Appl Physiol.

[41] Spencer M, Bishop D, Dawson B, Goodman C, Duffield R (2006). Metabolism and performance in repeated cycle sprints. Med Sci Sport Exerc..

[42] Pescatello LS (2014). ACSM’s Guidlines for Exercise Testing and Prescription.

[43] Flanagan EP, Jakeman PM (2006). Oral creatine supplementation and short-term dynamic power production in healthy young men. Int Symp Biomech Sport.

[44] Vincent WJ (1999). Statistics in Kinesiology.

[45] Cheillan D, Curt MJ-C, Briand G, Salomons GS, Mention-Mulliez K, Dobbelaere D (2012). Screening for primary creatine deficiencies in French patients with unexplained neurological symptoms. Orphanet J Rare Dis.

[46] Longo N, Ardon O, Vanzo R, Schwartz E, Pasquali M (2011). Disorders of creatine transport and metabolism. Am J Med Genet Part C (Seminars Med Genet).

[47] Abbiss CR, Quod MJ, Levin G, Martin DT, Laursen PB (2009). Accuracy of the Velotron ergometer and SRM power meter. Int J Sports Med.

[48] Astorino TA, Cottrell T (2012). Reliability and validity of the Velotron Racermate cycle ergometer to measure anaerobic power. Int J Sports Med.

[49] Clark NW. Wingate anaerobic test methods for power-trained males using velotron: Utah State Univesity; 2015. https://digitalcommons.usu.edu/gradreports/565/. Accessed 20 Apr 2016.

[50] Lopez E-ID, Smoliga JM, Zavorsky GS (2014). The effect of passive versus active recovery on power output over six repeated Wingate sprints. Res Q Exerc Sport.

[51] Nordeen-Snyder KS (1977). The effect of bicycle seat height variation upon oxygen consumption and lower limb kinematics. Med Sci Sports.

[52] Sanderson DJ, Amoroso AT (2009). The influence of seat height on the mechanical function of the triceps surae muscles during steady-rate cycling. J Electromyogr Kinesiol.

[53] Bini RR, Hume PA, Kilding AE (2014). Saddle height effects on pedal forces, joint mechanical work and kinematics of cyclists and triathletes. Eur J Sport Sci.

[54] Silberman MR, Webner D, Collina S, Shiple BJ (2005). Road bicycle fit. Clin J Sport Med.

[55] Korff T, Newstead AH, Van Zandwijk R, Jensen JL (2014). Age- and activity-related differences in the mechanisms underlying maximal power production in young and older adults. J Appl Biomech.

[56] Ericson MO, Bratt A, Nisell R, Arborelius UP, Ekholm J (1986). Power output and work in different muscle groups during ergometer cycling. Eur J Appl Physiol Occup Physiol.

[57] Havenetidis K, Cooke CB, Butterly R, King RFGJ (2006). Incorrect calculation of power outputs masks the ergogenic capacity of creatine supplementation. Appl Physiol Nutr Metab.

[58] Bouchard C, Tremblay A, Leblanc C, Lortie G, Savard R, Theriault G (1983). A method to assess energy expenditure in children and adults. Am J Clin Nutr.

[59] Green JM, McLester JR, Smith JE, Mansfield ER (2001). The effects of creatine supplementation on repeated upper- and lower-body Wingate performance. J Strength Cond Res.

[60] Izquierdo M, Ibanez J, Gonzalez-Badillo JJ, Gorostiaga EM (2002). Effects of creatine supplementation on muscle power, endurance, and sprint performance. Med Sci Sport Exerc..

[61] Burke DG, Silver S, Holt LE, Smith Palmer T, Culligan CJ, Chilibeck PD (2000). The effect of continuous low dose creatine supplementation on force, power, and total work. Int J Sport Nutr Exerc Metab.

[62] Powers ME, Arnold BL, Weltman AL, Perrin DH, Mistry D, Kahler DM (2003). Creatine supplementation increases total body water without altering fluid distribution. J Athl Train.

[63] Becque MD, Lochmann JD, Melrose DR (2000). Effects of oral creatine supplementation on muscular strength and body composition. Med Sci Sport Exerc..

[64] Hultman E, Söderlund K, Timmons JA, Cederblad G, Greenhaff PL (1996). Muscle creatine loading in men. J Appl Physiol.

[65] Greenhaff PL (1997). The nutritional biochemistry of creatine. J Nutr Biochem.

[66] Wyss M, Kaddurah-Daouk R (2000). Creatine and creatinine metabolism. Physiol Rev.

[67] Jeukendrup AE, Craig NP, Hawley JA (2000). The bioenergetics of world class cycling. J Sci Med Sport.

[68] Dotan R, Bar-Or O (1983). Load optimization for the Wingate anaerobic test. Eur J Appl Physiol Occup Physiol.

[69] Vandewalle H, Pérès G, Heller J, Monod H (1985). All out anaerobic capacity tests on cycle ergometers: A comparative study on men and women. Eur J Appl Physiol Occup Physiol.

[70] Robergs RA, Kennedy DD, Gibson DAL, Zuhl M, Hsu DH, Beam DJ (2015). Evidence for the invalidity of the Wingate test for the assessment of peak power, power decrement and muscular fatigue. Cent Eur J Sport Sci Med.

[71] Millet GP, Tronche C, Fuster N, Candau R (2002). Level ground and uphill cycling efficiency in seated and standing positions. Med Sci Sport Exerc.

[72] Reiser RF, Maines JM, Eisenmann JC, Wilkinson JG (2002). Standing and seated Wingate protocols in human cycling. A comparison of standard parameters. Eur J Appl Physiol.

